# A review of the state-of-the-art in automatic post-editing

**DOI:** 10.1007/s10590-020-09252-y

**Published:** 2020-12-24

**Authors:** Félix do Carmo, Dimitar Shterionov, Joss Moorkens, Joachim Wagner, Murhaf Hossari, Eric Paquin, Dag Schmidtke, Declan Groves, Andy Way

**Affiliations:** 1 Centre for Translation Studies, University of Surrey, Surrey, UK; 2 Department of Cognitive Science and Artificial Intelligence, Tilburg University, Tilburg, The Netherlands; 3 School of Applied Language and Intercultural Studies, Dublin City University, Dublin, Ireland; 4 ADAPT Centre, School of Computing, Dublin City University, Dublin, Ireland; 5 Microsoft, South County Business Park, Leopardstown, Dublin, Ireland; 6 ADAPT Centre, Dublin City University, Dublin, Ireland

**Keywords:** Automatic Post-editing, Neural Post-editing, Neural machine translation, State-of-the-art in Automatic Post-editing

## Abstract

This article presents a review of the evolution of automatic post-editing, a term that describes methods to improve the output of machine translation systems, based on knowledge extracted from datasets that include post-edited content. The article describes the specificity of automatic post-editing in comparison with other tasks in machine translation, and it discusses how it may function as a complement to them. Particular detail is given in the article to the five-year period that covers the shared tasks presented in WMT conferences (2015–2019). In this period, discussion of automatic post-editing evolved from the definition of its main parameters to an announced demise, associated with the difficulties in improving output obtained by neural methods, which was then followed by renewed interest. The article debates the role and relevance of automatic post-editing, both as an academic endeavour and as a useful application in commercial workflows.

## Introduction

Automatic Post-editing (APE) is an area of research aiming at exploring methods for learning from human post-edited data and applying the results to produce better Machine Translation (MT) output. Some possible uses of an APE system are:

- to improve MT output by exploiting information unavailable to the decoder, or by performing deeper text analysis that is too expensive at the decoding stage;
- to cope with systematic errors of an MT system whose decoding process is not accessible;
- to provide professional translators with improved MT output to reduce human post-editing effort;
- to adapt the output of a general-purpose MT system to the lexicon/style requested in a specific application domain. Bojar et al. (2017).

The usefulness of an APE system depends on how much it can add to a process which has already explored the best available methods and has produced the best possible output. Indeed, there are doubts as to whether it is a better strategy to implement an APE system or to fine-tune the MT system that produced the output with newly-available post-edited data. This and other questions will be discussed in the final sections of this article. It is important to note, at this stage, that APE can only be considered as a useful addition to an MT workflow if it exploits elements that have been omitted from the MT process, either by applying alternative technological approaches, or by incorporating new training data.

An APE project may focus on specific types of errors, it may dedicate a part of the research to text analysis, it may focus on the editing process, or it may implement specific linguistic resources. The capacity to present results that fulfil each of these purposes depends on the technological approach, which may be limited by available tools. A comparative study of APE methods by Chatterjee et al. (2015b) proposes that an APE project should also try to answer three questions:

- Does APE yield consistent quality improvements across different language pairs?
- What is the relation between the original MT output quality and the APE results?
- Which of the analysed APE methods has the highest potential?

These questions underline the research dimension of an APE project, since the capacity of a method to improve the quality of the MT output is only valid if it may generalise beyond its dataset. For research purposes, it is also important that publications look at the reasons why even minor improvements are achieved, so that different approaches can be used in new contexts and projects.

This review pays special attention to the APE shared tasks which have been held at WMT conferences since 2015, because these are privileged forums in which research teams test the most advanced technologies available each year, in comparable conditions. However, the article extends its reach to cover uses of APE beyond these shared tasks, namely by focusing on methods of evaluating APE, the connections between APE and other applications of MT, commercial uses of APE, and the connection to human post-editing, besides analysing the challenges and role of APE in a global context.

### Distinguishing between MT, APE and QE

The development of systems for MT, APE, and MT Quality Estimation (QE) shares some similarities, since all of these projects involve the implementation of full MT systems, or of some of their components. The most important input for all these systems is parallel data, also called ‘gold standard reference’, which contains aligned segments or sentences in two languages: one being the human translation (or target, TGT) of the other (the source, SRC). To establish the difference between these three types of systems, adapted to three different tasks, we will not dig more into their similarities.

In general, MT development requires parallel data^1^ to learn translation models (used to translate phrases, words or sub-word units from the SRC into the TGT language) and monolingual data to learn a language model (used to adapt the translation according to the TGT language properties). In neural machine translation (NMT), the translation and language models are usually trained jointly on the parallel data. From these models, an MT system generates and outputs a suggested TGT translation for each new SRC sentence. There is extensive research in testing new types of models and architectures in order to obtain better outputs, and MT systems may integrate optimisers and auto-correction components that fine-tune the results.

APE is focused on learning how to edit and improve the quality of the output of an MT system. To do that, rather than just providing the SRC data and the MT output, an APE system requires triplets of each segment, composed of:

- the source string (SRC);
- the version of that string in the TGT language, as produced by an MT system (identified simply as MT);
- and a version of that MT string after it was corrected by human post-editing (simply, PE).

In APE, the third element in the triplet should not be a reference translation (a human translation that was produced in a process in which MT was not present), since this would defeat the purpose of learning editing patterns for MT output, i.e. from what the human post-editors previously did to similar MT output. An APE system learns from these triplets what it takes to transform the MT output into a better version; it tries to estimate the transformation which occurred during post-editing, in order to generate better variants for the MT output of new source strings.

Given this, APE implies that there has first been a decoder producing the MT output, to which it adds a second decoder, producing an improved version of that output, so APE can be referred to as a process that involves “two-pass decoding” (Junczys-Dowmunt 2018a). Another aspect to take into account is that APE systems must identify words and segments that have been correctly translated by the MT system, so that it avoids editing these.

QE, in contrast, requires high quality data, be it post-edited MT output or human translations, which is used as gold standard reference, and annotations on the quality of that data. When these quality annotations are not available, QE systems produce their own labels, by identifying the features present in good and bad translations. These labels may be produced by a classifier that attributes “good” or “bad” tags to word alignments between MT output and post-edited sentences, or editing scores for each sentence, estimated with Translation Edit Rate (TER) (Snover et al. 2006). The QE system learns these scores and estimates the probable quality achieved by an MT system for new sentences. So, the purpose of QE is not to generate newly-translated sentences, but to estimate quality scores.

**Table 1:** Summary of main differences between MT, APE and QE tasks

	MT	APE	QE
Input	Parallel data	Triplets	Quality data
	(SRC+TGT)	(SRC+MT+PE)	(SRC+TGT+Score)
Purpose	To generate translations	To edit MT content,	To estimate the quality
	for new SRC sentences.	improving its quality.	produced by an MT system.
Output	New TGT content.	Edited TGT content,	Quality scores estimated
		with reduced editing scores.	for new SRC sentences.

The way these three tasks can be implemented in a complementary way in automated translation production workflows will be discussed in Sect. 6. For now, it is important to distinguish them as tasks with different purposes, different input data and different outputs. Table 1 summarises these details.

### Early methods for APE

Over the years, different methodologies have been used to tackle APE. The first references to automatic PE appeared in rule-based systems, as reinforcement of rules on specific issues created by these systems (Ryan 1988; Knight and Chander 1994; Allen and Hogan 2000). These systems often took the form of hybrid MT systems, which added statistical MT (SMT) capabilities to correct the results produced by commercial rule-based MT (RBMT) systems (Dugast et al. 2007; Lagarda et al. 2009). The use of statistical approaches based on the repetitive nature of errors produced by RBMT systems may also be observed in Simard et al. (2007a) and Simard et al. (2007b). The opposite strategy—the use of rules to correct SMT output—was also used, for example, to improve the results on morphologically-rich languages (Mareček et al. 2011; Rosa et al. 2012). PE rules based on patterns or problems identified in the target outputs are also still employed by researchers, such as Dowling et al. (2016), and by MT providers, e.g. KantanMT^2^.

Early APE researchers also looked for solutions which reapplied SMT techniques to correct specific problems in the output of their SMT systems. This created two-stage SMT architectures, where, after a first stage in which the SMT system created an MT hypothesis, a second “monolingual translation” stage occurred. In this second stage, a new system was trained on the output of the MT system and on the post-edited versions, both in the target language. The intuition was that the system would focus on the distances between the MT hypotheses and the references, eliminating errors that were created in the first stage.

In some of the experiments, researchers tried to maintain the connection to the source text, or the adequacy, as it is called. Béchara et al. (2011) applied a methodology that collected word alignments to the source, and they reported slight but inconsistent gains in lexical choice and reordering. However, most APE systems focused on fluency errors related to target-language issues that result from a lack of examples in the training data, a problem associated with data sparsity and out-of-vocabulary words. By training the systems for a second time on the MT output and the reference translations, the goal was to identify new connections between rare words and other more frequent ones, by manipulating the thresholds from which contextual information was retrieved. Parton et al. (2012) tackled specific linguistic adequacy errors, correcting them by either replacing or inserting words into the hypothesis. They used an automated system that retranslated whole sentences, incorporating corrections made by a rule-based APE system. This system fixed certain word-choice errors (e.g. numbers, names and named entities), but the methods of feeding corrections to the decoder had to be adapted for each new application.

Chatterjee et al. (2015b) discuss the two main methods available at the time for APE: monolingual, as in Simard et al. (2007a), in which a system performs a monolingual review of the MT output; and context-aware, as in Béchara et al. (2011), in which the system is reinforced by alignments to the source words and phrases, connecting resources in the two languages. The authors also stress that APE is especially important as means to feed corrections back to black-box systems.

Since 2015, there has been an upsurge in APE research, mainly as a result of the shared tasks organised at WMT conferences, which are discussed in the next section.

## The different ages of APE in WMT shared tasks

This section presents an overview of the different systems evaluated in five years of shared tasks at WMT conferences (2015 to 2019), highlighting the technological implications and potential of each. We analyse the publication of final reports of each shared task, and the most relevant papers published by the authors of the participating systems.

It is important to stress that this is not an exhaustive analysis of all publications about APE. We focus on the WMT shared tasks because of their relevance, not only related to the fact that they represent a testing ground for the newest technological approaches, but also because they provide comparable data that enables the advancement of evaluation methods.

Junczys-Dowmunt (2018b) called WMT15 the “stone age of APE”. In the same presentation, the author claimed that, in only 3 years, this task went from the bottom of the hill to the peak, with a “golden age” in 2017, to come back downhill fast, approaching its demise in 2018. We organise the next subsections according to the ages in this description, but we discuss the implications of this view and we extend it to 2019.

Table 2 presents a summary of the conditions and data that were provided to the teams that participated in five years of APE shared tasks.

**Table 2:** Summary of the initial conditions for WMT shared tasks 2015–2019

	2015	2016	2017		2018		2019	
Languages	EN–ES	EN–DE	DE–EN	EN–DE	EN–DE	EN–DE	EN–DE	EN–RU
Baseline	SMT	SMT	SMT	SMT	SMT	NMT	NMT	NMT
Domain	News	IT	Pharma	IT	IT	IT	IT	IT
Train set	11,272	12,000	11,000	25,000	28,000	13,440	13,442	15,089
Dev set	1000	1000	1000	1000	1000	1000	1000	1000
Test set	1817	2000	2000	2000	2000	1023	1023	1023
Extra data	N/A	N/A	4.5M	4.5M	19M	19M	19M	7.7M
RR-SRC	2.905	6.616	5.225	7.216	7.139	7.111	7.111	18.250
RR-MT	3.312	8.845	6.841	9.531	9.471	9.441	9.441	14.780
RR-PE	3.085	8.245	6.293	8.946	8.934	8.941	8.941	13.240
Zero edits	N/A	N/A	45%	14%	15%	25%	25%	61%

Over these 5 years, eight APE tasks were performed, but only four of them with the same language pair: English–German (EN–DE). In 2015, the APE task involved English–Spanish (EN–ES) training data, a type of content that was never used again (News), and training data that had been post-edited by the crowd. This was replaced in the 2016 shared task by data edited by professional translators, provided by the QT21 project (Specia et al. 2017). The characteristics of this corpus made it ideal for APE, being used ever since in the WMT shared tasks. In 2017, a second language pair was added (German–English: DE–EN) in the pharmaceutical domain. This content was also never used again. Finally, in 2019, a new language pair (English–Russian: EN–RU) was tested, with IT content provided by Microsoft.

In these tasks, different MT systems were used to create the output that had to be edited by APE. Participants in the competition never knew how systems were set up (black-box conditions), but the task reports presented the description of the MT systems that created the output. Until 2018, the black-box system was always a phrase-based SMT (PBSMT) system, but in that year a task with content created by an NMT system was tested for the first time. As of 2019, SMT output is no longer used for the APE task. The volumes of training data also increased over the years, especially with the availability of synthetic data. This will be described in Sect. 4.1. Table 2 also shows the number of segments in training, development and test sets, which did not change much over the years.

The repetition of words and phrases in the training data is one of the factors that is more closely associated with the capacity of APE systems to improve MT output (see below Sect. 2.3). Table 2 shows the repetition rates existing in the source side (RR-SRC), followed by the same rate for the MT output (RR-MT) and for the post-edited versions (RR-PE) included in the training data. This repetition rate is calculated as the geometric mean of non-singleton *n*-gram types (n=1–4), as suggested by Cettolo et al. (2014). As we can see, there is always an increase in repetition rate from the source language text to the MT output, but this is reduced by the human post-editing. The repetition rates of the EN–RU data used in 2019 are much higher than those for previous years, which may be related to the data selection process and the type of content: UI strings. Another factor associated with the results obtained by the APE systems is the number of segments that require no edits contained in the training and test datasets. We will comment on these in the sections below, but let us just highlight for now the salient values in the data for the 2017 DE–EN task (45% no edits), and for the 2019 EN–RU task (61%).

### WMT 2015: the stone age of APE

In 2015, a shared task on APE was held for the first time at WMT15 (Bojar et al. 2015, pp.28-36). This was the pilot run for APE shared tasks, with the main objective of identifying the state-of-the-art in the approach and an aditional purpose of setting a standard for the evaluation of APE systems in future competitions.

TER was adopted as the main metric for evaluation of APE. This metric estimates edit distances between two versions of a segment: the MT hypothesis and the human reference translation. In APE, the post-edited versions of the triplets are the references for the evaluations, and the output of each system is the hypothesis, with the distance between these two versions resulting in the TER score. This distance is an estimation of the minimum number of operations (deletions, insertions, substitutions and shifts of position of words), divided by the total number of words in a segment, that are required to transform one segment version into the other. The systems with the lowest TER are the best, since their output is closer to the references.

Besides applying TER as an automatic metric, the shared task evaluators also measured the precision of each system, in terms of the ratio of improved sentences (in which TER was reduced) over the total modified sentences.

The four research teams in WMT2015 presented SMT-based APE systems, with different components. One of the teams (associated with the Abu-MaTran project, and which made no specific publication for this participation) included a Recurrent Neural Network (RNN) classifier to classify words in the automatic post-edits as good/bad. The team from FBK (Chatterjee et al. 2015a) addressed data sparsity (most of the entries in the MT phrase table are unique) with a feature that measured the usefulness of each translated unit and pruned away the least useful ones. The team from LIMSI (Wisniewski et al. 2015) tried to develop sieves of rules that tackled known grammatical issues. Finally, Pal et al. (2015) tested different phrase lengths for the language and the translation model.

An unexpected result was reported for this experiment: none of the seven systems in the competition was able to improve on the baseline. This was associated with the challenges posed by the input data. The data analysis focused on data sparsity (measured as type/token ratio), and data repetitiveness (measured by singleton phrase pairs frequency). Bojar et al. (2015) mention the variability and inconsistency in crowd-sourced data as a probable cause for the results, together with the lack of repetition in news content.

A detailed evaluation showed that over-correction affected all systems, and that the systems that performed fewer edits were the most precise. The number of occurrences of each edit operation was also measured, but this analysis was not very informative.

For the next round of shared tasks, the authors of the final report suggested using professionally-edited in-domain data and multiple references for training, or a metric that would not penalise reasonable replacements of words. They also stressed the strong dependence on data repetitiveness/representativity. Finally, they suggested that researchers should focus on solving the tendency for systems to over-correct.

### WMT 2016: first neural APE systems

In the shared task at WMT16, the dataset was composed of sentences from the IT domain, edited by professional translators, and the language pair was EN–DE. The evaluation now also included BLEU (Papineni et al. 2002), which has the added advantage of dealing with *n-*grams.

This year, APE methods became more sophisticated, incorporating neural, log-linear, and factored models (Bojar et al. 2016). The following summary presents the systems by their ranking based on TER:

*AMU*: The submission by Adam Mickiewicz University (Junczys-Dowmunt and Grundkiewicz 2016) was one of the first to employ neural models in APE. It also innovated in the use of synthetic training data (described in Sect. 4.1), in the use of a log-linear combination of monolingual and bilingual models to create an ensemble, and the addition of a feature to control the final quality (this is an adequacy control feature that penalises words which appear in the output but do not appear in the input). The best results from simple models in this paper are obtained with monolingual models (trained on MT–PE). From these simple models, the ensembles explored the capacity of NMT systems to accept more than one input, in this case, monolingual and bilingual models, a method which increased the precision of the system by about 2 TER and BLEU points. This strategy was to be known as “multi-source” (although “multi-input” might be a more accurate description). This method was thoroughly explored in subsequent APE systems.

*FBK*: The submission by Chatterjee et al. (2016a) was basically a combination of previous SMT approaches in a factored model: a monolingual one, which had a high recall, and a more precise context-aware variant. However, the system also included neural language models and a QE model which was intended to select the best translation between the MT output and the APE output. As we will see in Sect. 4.2, the incorporation of QE features is a strategy that was successfully used by more recent systems. To avoid over-correction, the authors implemented a data selection process that prevented segments above a certain threshold of similarity being edited.

*Saarland University*: The system presented by Pal et al. (2016b) used a combined PBSMT system with a model called OSM (Durrani et al. 2011). This method represents the post-edited translation process as a linear sequence of operations that take into account previous context and seemed to be able to model very well reordering requirements, particularly for German.

*Jadavpur and Saarland universities*: This system (no publication) took a phrase-based approach and focused on two specific types of errors: presence of unwanted words and word surface errors.

*DCU*: Dublin City University’s submission (no publication) was designed as an automatic rule-learning system. The system learned four editing actions (replacement, deletion, insertion, and reordering) from alignments, with no linguistic knowledge involved. It then recorded alignment pairs and their contexts in the source and target sentences and extracted replacement rules, the precedence ordering of those rules, and the maximum number of rules in a sentence.

*CUNI*: The system from the Univerzita Karlova v Praze (Libovický 2016) was also an NMT model with attention. To improve the capacity of the system to focus on edits, the target sentence incorporated a sequence of edit operations, namely a keep, a delete, and an insert operation. The system had a mechanism of pre- and post-processing to deal with specific issues for German related to articles, prepositions, and pronouns.

The authors of the report on the shared task mention that neural APE (or simply NPE) may have a better capacity to deal with data sparsity and the overfitting which may result from the methods used to create synthetic data. A question remained as to whether this was due entirely to NMT solutions, or to the amount and features of the training data, namely repetitiveness and consistency. Over-correction still affected many systems in the shared task.

A detailed analysis of the results showed the higher precision of the top system (the AMU system, where 58% of modified sentences were improved). This system also showed a higher capacity to make correct shifts of position of words, a capacity which, as hypothesised in the shared task report, might spring from NMT’s capabilities. It is also the most balanced system in terms of distribution of editing actions, very close to the actual distribution of edits in the baseline data.

Finally, it was decided to do a human evaluation stage of the results of the APE systems, using a ranking system based on Appraise (Federmann 2012). This evaluation showed a high correlation between the human evaluation and the automatic metrics, confirming the neural approach of the AMU APE system as the one yielding the best results, albeit still a long way behind human post-edits.

Table 3 presents a comparison of the results obtained by the best APE systems in the first two years of the WMT shared tasks. The table contains the values of both TER and BLEU, for the baseline and the best APE system, followed by the estimate of gain (or loss) achieved by the APE system. A negative value for the difference in TER means an improvement in reducing the editing effort, whereas a positive value for the difference in BLEU shows an increased similarity with the human post-edits. The table shows that the best APE system in 2016 improved over the baseline both in TER and in BLEU, while the best APE system in 2015 was not able to beat the baseline in the only metric that was used.

**Table 3:** Summary of the evaluation of the best APE systems in WMT shared tasks in 2015 and 2016

Year	2015	2016
Language pair	**EN–ES**	**EN–DE**
Best APE system	FBK	AMU
MT+APE technology	SMT+SPE	SMT+NPE
Baseline: TER (mt,pe)	22.91	24.76
Best APE: TER (ape,pe)	23.23	21.52
**Difference**	**0.32**	**− 3.24**
Baseline BLEU	N/A	62.11
Best APE BLEU	N/A	67.65
**Difference**	N/A	**5.54**

### WMT 2017: the golden year of APE

The third run of the APE shared task was the most successful one, with virtually all systems obtaining results above the baseline (Bojar et al. 2017). All systems tested in WMT17 were neural-based end-to-end solutions and involved multi-source models. Two language pairs were tested. The next paragraphs describe the systems submitted to the shared task, by their ranking for the EN–DE pair.

*FBK 2017* Chatterjee et al. (2017a): This is a multi-source ([SRC+MT] -> PE) NMT approach with an ensemble of 8 models. The NMT system had two bi-directional encoders—one for the SRC and another for the MT—each having its own attention mechanism computing a weighted context. The final (joint) context vector was obtained through a merger layer where the contexts of the SRC and of the MT were concatenated and a linear transformation was applied. The final context was used to produce the final APE strings. The data used was synthetic and authentic. To improve the performance of the APE system, the authors used context dropout: a shared dropout to the hidden state of both encoders and a dropout on the merger layer. The authors built 4 types of networks, and then ensembled the 2 best models of each type. To improve the performance further, the systems employed a task-specific loss (TSL) function that took into consideration the attention score of the MT words before computing the target probability. The resulting types were: SRC-PE, MT-PE, SRC-MT-PE, SRC-MT-PE-TSL. Further, they optimised the weights of the ensemble using batch-MERT (Och and Ney 2003) which improved their scores. They also incorporated two rerankers—one with shallow features and another with statistical features. The resulting systems led to improvements of + 7.6 BLEU and − 4.9 TER (for EN–DE).

*AMU-UEdin 2017* (Junczys-Dowmunt and Grundkiewicz 2017): Aside from a single-source system (in which either SRC or MT is trained against the PE), this project used multi-source APE, by combining SRC and MT as the input and the PE as the output. To train their APE system, the authors generated additional synthetic data using round-back translation with PBSMT systems. The interesting aspect of this work is that they employed different types of attention, as well as GRU networks. They used: (i) a reimplementation of the standard attention mechanism (Bahdanau et al. 2015) with conditional GRUs; (ii) hard monotonic attention: (Aharoni and Goldberg 2017); and (iii) soft double-attention: doubly-attentive decoder for multimodal NMT to cover the SRC and MT contexts (Calixto et al. 2017). As a final system, they provided an ensemble of four NMT systems that combines the hard and the double attention mechanisms. Each model followed from distinct training runs with different random weights initialisations.

*DCU 2017* (Hokamp 2017): This is an ensemble of five neural models that jointly aim to produce the best post-edited variant of MT sentences. Details of the five model types follow:

- the first model type is trained on the aligned MT and PE data;
- the second is trained on a concatenation of source and MT (SRC|MT) as input, and PE data as output, with one encoder;
- the third is a SRC+MT-factor model, in which features such as PoS or dependency relations are integrated on the MT side;
- the fourth model extends the previous one with word-level tokenisation, using byte-pair encoding (BPE);
- for the fifth model type, four ensembles of NMT models are created: the first ensemble results from the averages of the best check-points of each model type; the second ensemble is tuned on TER; the third ensemble is tuned on F1-Mult; and the fourth is an ensemble of 8 models in total, bringing together four individual SRC models and the averaged models from each model types.

To determine the weights for each model in the ensemble, the system is tuned with respect to TER. This approach combines several techniques that improve the performance of the NMT models, such as: (i) including features (or factors) during training; (ii) using word-segmentation, i.e. BPE (Sennrich et al. 2016c), to overcome out-of-vocabulary issues; and (iii) incorporating word alignments. In addition, models that take a combination of SRC and MT as input, instead of two encoders, concatenate the input vectors and indicate the end of the source and the beginning of the MT with a special token (|). This allows the use of a standard NMT encoder-decoder architecture with bidirectional RNNs and an attention mechanism, rather than needing to build a new, more complex, system. The particular interest in this system is driven by the concatenation process, the distributed approach and the ensembling of multiple NMT systems.

This system was also trained as a QE system, and this relation is discussed in Sect. 4.2.

*USAAR 2017*: There is no specific publication which describes this system. However, the shared task report allows us to highlight a few features from the system. It compared a neural model against an OSM, which is based on corpus processing, hybrid word alignment and PBSMT. The NMT model was better for segments with up to 15 words, but the OSM was better for longer ones, which may be related to NMT’s problem of coverage for longer sentences (Koehn and Knowles 2017). The final submission was a combination of NMT for shorter sentences and OSM for longer ones.

*LIG 2017* (Bérard et al. 2017): Although this was not one of the best-classified systems, their models deviated from the other approaches and presented some interesting reflections on how to learn the editing processes. The authors’ approach did not focus on learning word patterns but on learning four edit operations: keep, delete, insert and end of sentence, but it did not incorporate shifts (adding substitution as an edit symbol would increase data sparsity). The NMT model learned a global attention model, with a decoder that incorporated a hard attention mechanism. It used a chained architecture which combined two encoder-decoder models, one to align SRC and MT, and one to learn the MT-PE edits. The latter system used forced attention over the MT sequence, as well as the attention vectors over SRC computed by the first system. The models were trained jointly. When the system needed to predict an edit operation on a word, it looked at the MT word to post-edit, and at the SRC word it aligned with. The analysis of the results shows that the system presented the best results very early in the training phase and then it started to overfit. The authors identified an imbalance in the operations: keep is over-represented, which results in the system adopting a very conservative approach, since the other symbols appear few times in the data. They suggest using weight sharing or multitask training to overcome this effect, admitting that the method used to extract the edits is artificial and does not correspond to the actual operations performed by the post-editor. They also discuss whether edits occur at character level and how this would affect the volume and sparsity of data.

*JXNU 2017* (Tan et al. 2017): This proposal was based on learning edits from the dataset with a neural post-editing system, and then used a sentence-based QE system to rank the best outputs and select the best model. The decision to base the system on learning edit operations comes from the observation that most of the segments in the data required between 1 and 4 edits to be corrected. The data was then stratified by extracting three separate corpora according to the number of edits required. In the training, the authors also used synthetic data and subword units. The authors’ analysis of the results shows that the system had been able to contain the over-correction tendency. However, the focus on segments with a low number of corrections may have affected the capacity of the system to learn the edits in more complex editing schemes.

*CUNI 2017* (Varis and Bojar 2017): The research that led to the CUNI system submission focused on two main topics: multi-source input and subword tokenisation based on units or characters. To address multi-source input, the authors explored the potential of a single-encoder versus a double-encoder. The former method considers the concatenation of the SRC and MT sequence as input to a single encoder RNN: the RNN encodes the SRC and the MT sequences in a common embedding space. Using an attention mechanism allows mitigation of the requirement of a larger vocabulary and the difficulties with longer sequences. For the single- and double-encoder systems, the authors used dictionaries based on either subword units or single characters. The evaluation showed that the best architecture (the concatenated character-based one) actually degraded the BLEU score of the MT output. It only achieved an improved BLEU score after the data was increased with synthetic data. A manual examination of the results showed that the system was taking a conservative approach and keeping most of the MT output unchanged. The authors mention that BLEU (and automatic metrics) do not give a reliable picture of PE performance, since only in the manual evaluation did they notice that, although their system helped with main verbs, it also damaged the sentence structure and introduced spelling errors. Finally, although the character-to-character concatenated systems achieved the best results in the experiments, the authors decided to submit the two-encoder system, so as to keep the explosion of data under control.

*Results*: Table 4 below shows the results obtained by the best systems in the two language pairs. The TER and BLEU scores of the baseline data were very different for the two datasets, with DE–EN showing an impressive 15.55 TER and 79.54 BLEU, while for EN–DE the corresponding values are 24.48 and 62.49, more in line with the previous year’s scores. These factors were associated with the capacity of APE to improve the MT output. However, when there is a low repetition rate and low editing, the APE systems seem not to be capable of gaining much. A closer look at the training data shows that the high average TER and BLEU scores of the DE–EN data are related to a high number of segments not requiring any edit (about 45% of the segments, cf. Table 2). This high number of non-edited segments calls for very precise APE systems, which should be capable of not editing these segments. It is no surprise that only in the EN-DE language pair did systems actually achieve promising results.

**Table 4:** Summary of the evaluation of the best APE systems for the two tasks in WMT 2017

Year	2017	
Language pair	DE–EN	EN–DE
Best APE system	FBK	FBK
MT+APE technology	SMT+NPE	SMT+NPE
Baseline: TER (mt,pe)	15.55	24.48
Best APE: TER (ape,pe)	15.29	19.60
**Difference**	**− 0.26**	**− 4.88**
Baseline BLEU	79.54	62.49
Best APE BLEU	79.82	70.07
**Difference**	**0.28**	**7.58**

The results of all submissions show many systems with improvements of at least 4 TER points and 5 BLEU points over the baseline (the best system—FBK—achieved 19.60 TER and 70.07 BLEU) in EN–DE (see Table 4). This was a confirmation of the success of neural approaches to APE (NPE).

### WMT 2018: the last year of useful APE?

The report of one of the teams which participated in the APE shared task in WMT 2018 concluded: *We further believe that this might constitute the end of neural automatic post-editing for strong neural in-domain systems. The next shared task should concentrate on correcting general domain on-line systems. Another interesting path would be to make the original NMT training data available so that both pure NMT systems and APE systems can compete. This would show us where we actually stand in terms of feasibility of neural-on-neural automatic post-editing.* (Junczys-Dowmunt and Grundkiewicz 2018, p. 838)

The main reason behind this type of statement is the new challenge proposed for the shared task: to use the same technology for both NMT and APE, i.e. NPE to improve on NMT output. The very small improvements obtained in such circumstances by all competing systems led to a frustrating tone in the findings report of the task (Chatterjee et al. 2018a). This result even overshadowed the fact that NPE on SMT output represented a very positive evolution over the previous year’s results.

The WMT 2018 challenge included an APE sub-task on SMT output and another one on NMT output. Although the language pair and domain were the same for the two sub-tasks (EN-DE IT content), the volumes and features of the training and testing data were very different. For the SMT sub-task, 28,000 triplets were available for training, whereas only 13,000 triplets for the NMT sub-task (see Table 2).

Table 5 shows how SMT training data has worse scores in TER and BLEU metrics than NMT output (24.24 TER and 62.99 BLEU for SMT, against 16.84 TER and 74.73 BLEU for NMT). As may be observed in Table 2, repetitiveness rates for both datasets were very similar, and as high as expected for IT content. Nevertheless, there is a major difference from the two outputs, which is the percentage of segments that required no editing (i.e. which have TER=0): 15% of the total segments in the SMT output did not require any editing, compared with 25.2% of segments in NMT output. This meant that there were more segments that should not be edited in this output, and APE systems had fewer editing patterns to learn from, which made this a tougher challenge for the competing systems.

We also ought to note that in this shared task, besides the synthetic data created for WMT2016, a new dataset of synthetic data for training APE systems was introduced: the eSCAPE synthetic corpus (Negri et al. 2018b) (see Sect. 4.1). This dataset is composed of two sets of 7,258,533 EN–DE synthetic triplets each, one generated with PBSMT and the other with NMT.

We present below the five competing systems, following the ranking order of the systems according to the main metric on the SMT systems. With the exception of one of the submissions by the German Research Center for Artificial Intelligence—MLT group (DFKI–MLT), the five systems used Transformer-based architectures (Vaswani et al. 2017).

*MS-UEdin*: The system presented by Microsoft and the University of Edinburgh (Junczys-Dowmunt and Grundkiewicz 2018) used the artificial datasets and the original training data provided by the organisers, oversampled 20 times. The APE system they developed used the original Transformer configuration. It extended the original architecture by adding one more encoder and stacking an additional target-source multi-head attention component above the previous target-source multi-head attention component. In addition, following their own insights from previous tasks, they tied the embeddings across all encoders and shared all parameters, despite the fact that they encode different languages. A major part of the synthetic data available was split into subsets using domain-selection algorithms aimed at isolating useful portions of the IT domain; these subsets were used to train different models, which then produced the submission via an ensembling process.

*FBK* :The system by FBK also employed a multi-source transformer in their APE submissions (Tebbifakhr et al. 2018). They followed the approach adopted in Chatterjee et al. (2017a) for their submission to the previous APE shared task, but with a transformer-based encoder/decoder. A more interesting point in their work is the use of a risk function for optimising the model at the sentence level rather than on the token level, as usual with a maximum likelihood estimation (MLE) loss function. This approach follows the minimum-risk training (MRT) introduced by Shen et al. (2016). Their submissions included an MRT and a linear combination of MRT and MLE systems. Trained on all the data provided (original and synthetic) with additional preprocessing on the German side, the MRT submission ranked first in the NMT subtask and third in the PBSMT subtask (the MRT+MLE ranked second in the PBSMT subtask).

*POSTECH*: The system by Pohang University of Science and Technology (Shin and Lee 2018) is an extension of the transformer architecture with an additional encoder and with two multi-head attention layers to the decoder: one for the original translation dependency (${\text{SRC}} \to {\text{MT}}$) and another for the ideal translation dependency (${\text{SRC}} \to {\text{PE}}$). The latter extension aims to define a dependency between the common words in the MT and PE texts and the SRC text, so that those words obtain a similar source context. The synthetic data was divided into different portions, extracted in step-wise data reductions. The final submissions selected results from the best systems and combined them with different ensembling techniques.

*USAAR-DFKI*: Saarland University and DFKI participated with another multi-source transformer system (Pal et al. 2018). There are two points that draw research interest. Firstly, they implemented a three-encoder architecture where one encoder reads and encodes the source, another reads and encodes the MT output, and a third one takes as input the concatenation of the output of the first two and produces a representation that is used during decoding. Secondly, they ensembled their models’ output based on the frequency of commonly-produced words. Furthermore, they also used a single source transformer model trained on MT-PE pairs. For the PBSMT subtask they ensembled a single source with a multi-source model; for the NMT subtask they ensembled five models: three single source models, one of which was additionally fine-tuned on a subset of the training data, and two multi-source models, one of which was also fine-tuned on a subset of the data.

*DFKI-MLT*: DFKI submitted LSTM- and transformer-based systems (Pylypenko et al. 2018). While they used standard implementations for their systems—OpenNMT (Klein et al. 2017) for the LSTM system and Marian (Junczys-Dowmunt et al. 2018) for the Transformer systems—they aimed at boosting their performance by jointly training on the NMT and PBSMT data. To distinguish between the different data origins (NMT or PBSMT) and data types (i.e. original or synthetic), the data were augmented with a prefixed token indicating its source. However, these conjoined systems did not yield good results as their submissions were ranked last. The submissions used two neural architectures: an attentional RNN with gated units and a multi-head attention-only network.

*Results* The results of all systems were quite promising in the SMT subtask, with three submissions out of eleven outperforming the baseline by at least a 5-point reduction in TER and an 8-point increase in BLEU. The best system (MS/UEdin Primary) achieved an improvement of more than 6 TER and almost 10 BLEU points (see Table 5). The authors of the findings report commented that these positive results might be due to either the technological advances in the systems or to the addition of the extra synthetic training data.

**Table 5:** Summary of the evaluation of the best APE systems for the two tasks in WMT 2018

Year	2018	
Language pair	EN–DE	EN–DE
Best APE system	MS/UE	FBK
MT+APE technology	SMT+NPE	NMT+NPE
Baseline: TER (mt,pe)	24.24	16.84
Best APE: TER (ape,pe)	18.00	16.46
**Difference**	**− 6.24**	**− 0.38**
Baseline BLEU	62.99	74.73
Best APE BLEU	72.52	75.53
**Difference**	**9.53**	**0.80**

However, the same systems did not yield improvements above one TER or BLEU point when applied to NMT output. The best system in this subtask, (FBK Primary, MRT), improved the TER score by only 0.38 and BLEU by only 0.8. This was considered in line with those achieved in the previous year’s shared task, on output that had similar initial good TER and BLEU scores (see Table  4). Due to this, the authors of the report suggest that the quality of the MT output plays a more important role on the results achieved by APE than the technology employed.

The results of the different APE systems in the 2018 shared task were very close to each other, one reason being that none brought a novel way of performing APE. Despite some interesting approaches, the different methods used to explore the Transformer architecture and the synthetic data did not widen the technological landscape in APE. Moreover, since the baseline systems were not trained on the same data and since the amount of training data for the NMT subtask was smaller than for the PBSMT task, it is not possible to fully judge why post-editing NMT does not yield the same performance gain found in the PBSMT subtask.

### WMT 2019: can NPE run the last mile?

After 4 years of WMT shared tasks on APE, the main question for 2019 was a tough one: could APE prove its worth, by improving the small margin that high-quality NMT outputs produce? As Table 2 shows, the organisers decided to use the same EN-DE dataset that was used in WMT 2018, to check how much the new proposals were able to improve on what was considered to be a disappointing outcome. In addition, participants were challenged with a more demanding language pair (EN–RU), which only included segments that did not require heavy editing. The suggestion was that this data would test the capacity of each system to only perform the minimal amount of editing necessary to improve on a high-quality MT output.

Chatterjee et al. (2019) report on the findings of this shared task. They note that three main techniques are shared between the seven teams that participated in WMT 2019: (i) exploration of the Transformer architecture, (ii) a multi-source approach and (iii) the use of synthetic data. However, there are other methods and approaches that are shared between the participating systems, as we will see below.

Lopes et al. (2019) describe the system developed by Unbabel which also achieved the best results for the EN–DE pair in 2019: an encoder-decoder framework, strongly based on BERT (Devlin et al. 2019), a bi-directional language representation model based on Transformer that has been tested in many applications and has achieved remarkable results. It was used for the first time in APE by Unbabel, but the FBK team also applied it as a fine-tuning component (Tebbifakhr et al. 2019). Instead of a multi-encoder system, Unbabel used a concatenation of SRC and MT, with a BERT token, which is fed to an encoder-decoder architecture. Unbabel also applied a penalty to constrain decoding so as to be as close as possible to the input as a way to avoid over-correction, and during training it used in-domain data provided by another shared task. This last data was also used by the submission of the University of Sheffield & Imperial College London. Almost all teams describe using the eSCAPE corpus for training, but the POSTECH team mentions that it filtered the data in order to obtain similar statistics to the ones in the task training data. The same team used the adjustment of ‘teacher-forcing ratios’ to alleviate the exposure bias (Lee et al. 2019). Another technique exploited by the systems in 2019 is the addition of tokens that identify different partitions of the training data. FBK, for example, used tokens that identified the amount of editing required by the segments, divided into three levels: no editing, light editing and heavy editing. The team from ADAPT/DCU presented two very different proposals, one guided by the addition of these tokens (one related to the segment length and the other related to the topic), and a second approach based on interleaving two different MT technologies. Exploring an inversion of the past strategy of interpolating two MT technologies (when NMT was used to improve SMT output), the team decided to test the effects of using an SMT model to improve NMT output (Shterionov et al. 2019).

Table 6 shows the results obtained by the best systems for the two language pairs (EN–DE and EN–RU) in WMT 2019.

**Table 6:** Summary of the evaluation of the best APE systems for the two tasks in WMT 2019

Year	2019	
Language pair	EN–DE	EN–RU
Best APE system	UNBABEL	ADAPT/DCU
MT+APE technology	NMT+NPE	NMT+NPE
Baseline: TER (mt,pe)	16.84	16.16
Best APE: TER (ape,pe)	16.06	16.59
**Difference**	**− 0.78**	**0.43**
Baseline BLEU	74.73	76.20
Best APE BLEU	75.96	75.27
**Difference**	**1.23**	**− 0.93**

The best EN-DE system reached a TER which was only − 0.78 TER and + 1.23 BLEU better than the baseline. Aside from this system, three more achieved better results than the systems in 2018. This is a good indication that the new NPE approaches may be worth further exploration, with the BERT fine-tuning models playing an important role here.

The results in EN–RU were disappointing: there were only three competing systems and none beat the baseline. This was not surprising, since the morphological richness and data sparsity of this language pair present big challenges for any MT system. Furthermore, given the high-quality of the dataset—the ratio of segments that required no edits was an impressive 61% (see Table  2)—and the fact that it mainly contains short strings, the difficulties in improving the output were to be expected. It was a surprise, though, that despite not being able to improve on the baseline system, the SMT APE system from ADAPT/DCU was the one to achieve the best results. This raises a question on the role of using hybrid strategies in APE: is this strategy still valid when such an approach implies retrieving older technologies? The specific conditions of this subtask do not help answer this question, so it would be interesting to see this tested in more comparable conditions.

## Evaluating the state-of-the-art in APE

Over 5 years, APE shared tasks travelled a long way, from the pilot experiments in 2015, to the challenging environment of 2019. During this period, the methods used to evaluate the performance of APE systems also evolved. In this section, we highlight the methods used to evaluate APE in the state-of-art environment, namely in the last 2 years, with APE systems trying to improve high-quality NMT output.

The organisers of the shared tasks in WMT conferences have been meticulous in analysing the features of the data that might influence the expected results. One of the main contributions of shared tasks is testing and helping the development of evaluation methods adjusted to the requirements of the task. Aside from automatic metrics (TER and BLEU), APE systems have always been evaluated in terms of precision (number of improved sentences over total edited sentences). Since 2018, in the context of NPE applied to NMT output, a new form of evaluation against reference translation has been attempted, to try to grasp the effect of penalisation of acceptable corrections and of over-correction. Finally, direct assessment (DA) (Graham et al. 2013) has been applied since 2017 as a standard form of human evaluation (see Sect. 3.5). In this section, we analyse the impact of these forms of evaluation on the knowledge the scientific community has gained from the APE shared tasks.

### Automatic metrics

TER and BLEU are the main metrics used to evaluate APE systems. TER’s decomposition into clear operations that describe the editing process in terms akin to the actual edits made by humans enables detailed analyses, such as the one that shows that when systems are not balanced in terms of the typical proportion of editing operations in the training data, they tend to have bad overall performance. The addition of BLEU did not contribute to a better understanding of APE, with only occasional system rankings being different between the two metrics. The results presented by these two metrics have been sufficiently analysed in previous sections in this article, so we will move on with the analysis of the other forms of evaluation used in the APE shared tasks.

### Evaluating style and over-correction penalisation

Since TER and BLEU are computed against one single post-edited version per sentence, any divergences from that reference, no matter how correct they may be, will be penalised. Because of that, the evaluators of the 2018 shared task decided to add an evaluation process against external independent references. It was the first time that this type of evaluation was applied in an APE shared task, but it is not clear whether this form of evaluation has demonstrated its value.

The authors of the 2018 report explain that the purpose of this evaluation was twofold: (i) to measure the capacity of the APE systems to capture the style of the PE versions, and (ii) to identify over-corrections created by the APE systems. The methods designed to accomplish these two purposes look for the following relations:

- The gains of APE systems over baselines should be higher when TER and BLEU scores are measured against the original human post-edits in the training data than when the same scores are calculated against independent human reference translations. This would indicate that the APE systems had learned particular features of the human PE process, which the authors call ‘style’.
- After estimating combined TER and BLEU scores, using both human post-edits and independent human translations, one can compare these combined scores with the same scores obtained by the APE systems and baseline systems against the post-edits alone. Higher combined TER and BLEU scores are interpreted as containing acceptable alternative translations that were not present in the post-edits. This would show the tendency of the automatic metrics to penalise good edits, and these are classified as ‘over-corrections’.

The WMT reports do not describe how the reference translations were collected, but it is assumed that these are part of the QT21 corpus^3^, which means that there is one alternative translation for each sentence, produced by human translators with no access to MT output. It is also not described how the TER scores are estimated; a simple notation like TER(npe,hpe)-TER(mt,hpe) would help identify how the gains of the APE systems over the baseline MT output are estimated. This is the estimation that yields the values shown in the second row of Table 7. The third row in the same table is estimated as TER(npe,href)-TER(mt,href). We would also suggest that the independent translations should not be identified simply as ‘references’, as all TER scores have hypotheses and references; we suggest the use of ‘href’ instead, for ‘human reference’. Besides, the methods to combine several references in TER and BLEU scores have been shown to be complex, with implications over the conclusions we may take from their use. Dreyer and Marcu (2012) propose a variation of TER, named HyTER, to deal with this challenge, while Qin and Specia (2015) discuss the complexities of multiple references in other metrics. We assume that the authors applied the process described by Snover et al. (2009), who explain that each reference is scored individually against the same hypothesis, then the reference with the fewest edits is used as the numerator, and the average number of words in all references is used as the denominator. With only one human post-edit and one unconstrained human translation per sentence, it is arguable whether one can build interpretations based on a score of multiple references. Using the lack of overlap of this multiple-reference score with the scores of the post-edits as a tool to estimate the sensitivity of APE systems and automatic metrics to over-correction may also be considered unwarranted.

Tables 7 and 8 illustrate the results obtained in these evaluations, in the three tasks in which they were used: in 2018 for SMT output and in 2018 and 2019 for NMT output, always with the same dataset.

**Table 7:** Results of the evaluation of style sensitivity using external independent translations

	2018 (SMT+NPE)	2018 (NMT+NPE)	2019 (NMT+NPE)
Best APE system	MS/Uedin	FBK	Unbabel
TER gains w/ HPE	− 6.24	− 0.38	− 0.78
TER gains w/ HREF	− 5.67	0.02	− 0.58
**Style sensitivity (TER)**	**0.57**	**− 0.40**	**− 0.20**
BLEU gains w/ HPE	9.53	0.80	1.23
BLEU gains w/ HREF	6.51	0.08	0.75
**Style sensitivity (BLEU)**	**3.02**	**0.72**	**0.48**

**Table 8:** Results of the evaluation of penalisation of over-correction using external independent translations

	2018 (SMT+NPE)	2018 (NMT+NPE)	2019 (NMT+NPE)
Best APE system	MS/Uedin	FBK	Unbabel
TER(hpe)-(multi) Best APE	0.97	0.43	0.48
TER(hpe)-(multi) Baseline	0.48	0.57	0.57
**Over-penalisation (TER)**	**0.49**	**− 0.14**	**− 0.09**
BLEU(hpe)-(multi) Best APE	− 4.18	− 1.83	− 2.14
BLEU(hpe)-(multi) Baseline	− 3.22	− 2.10	− 2.10
**Over-penalisation (BLEU)**	**− 0.96**	**0.27**	**− 0.04**

The tables of results show very low values for both the style sensitivity evaluation method and for the penalisation of over-corrections. The 2018 evaluation of APE of SMT output might point to a promising result, but in the context of NMT, these methods proved to be frustrating. One of the comments in the reports admits that *“Though minimal, these differences suggest that a certain amount of corrections made by the top systems still represent acceptable modifications of the original translations.”* (Chatterjee et al. 2019 p. 21). The reports of both shared tasks fail to convince on the usefulness of this form of evaluation: the methods are not clear enough, the assumptions and interpretation of results are not solidly grounded, and the results are not strong enough to advise its exploitation outside the scope of the shared tasks.

### Precision

The evaluation of precision is an interesting one, which deserves more exploration. The reports of the shared tasks often discuss the averages of all systems, since their purpose is ranking systems competing in similar conditions. In Table 9 we look at the results from the best systems in all WMT APE events. In this table, ‘coverage’ means the number of modified segments over the total test set, ‘precision’ is the percentage of segments in which there was a reduction in TER over all modified segments, and ‘deterioration’ the ratio of segments in which TER was higher, showing that the editing effort had increased.

**Table 9:** Results of the evaluation of precision in all shared tasks

Year	2015	2016	2017		2018		2019	
Language pair	EN–ES	EN–DE	DE–EN	EN–DE	EN–DE	EN–DE	EN–DE	EN–RU
Best APE	FBK	AMU	FBK	FBK	MS/UE	FBK	UNBABEL	ADAPT/DCU
MT+APE	SMT	SMT	SMT	SMT	SMT	NMT	NMT	NMT
	+ SPE	+ NPE	+ NPE	+ NPE	+ NPE	+ NPE	+ NPE	+ NPE
Coverage	15%	80%	13%	80%	**82%**	27%	36%	9%
Precision	23%	58%	40%	64%	**68%**	47%	**51%**	18%
Deterioration	53%	23%	29%	**21%**	**21%**	28%	30%	53%

The most precise systems were those presented in 2018, especially in the task in which SMT output was edited by NPE. The best system achieved a precision of 68%. This is also the system that edited more sentences, a total of 82% of all sentences in the training data. No system has yet been able to achieve this precision with NMT content. The most precise system for this type of content in this table was the best performer in 2019: the system by Unbable improved 51% of the sentences it modified. However, in the same year, there was a more precise system: the system presented by POSTECH, which is not represented in this table but improved 61% of the edited sentences. This system also only deteriorated 20% of the edited sentences, whereas Unbabel’s increased the editing scores of 30% of the edited sentences. It is important to note that the POSTECH system was very conservative, with only 20% of all sentences modified.

As we have commented before, the ability to control over-correction is a requirement for APE systems. In Table 9, all systems deteriorate at least 21% of the sentences, despite all efforts to tackle this effect. A more in-depth analysis of these precision figures, which could be related to an analysis of the patterns of mistakes created by each system, could help improve the behaviour of the APE systems. The next section comments on a method of evaluation that looks into such patterns.

### Edit operations

The analyses of distribution of edit operations (deletions, insertions, substitutions and position shifts), as measured by TER, has revealed a very regular behaviour of the best-performing APE systems. All systems perform more substitutions than any other operation (around 40% in 2018 and 54% in 2019). The next most common operations are deletions (around 20%), insertions (closer to 15%) and finally shifts (usually below 10%). This distribution is common even in human PE, but it is also related to the process of estimating TER (do Carmo 2017). APE systems that are poor performers usually show a very different behaviour, usually with a higher preponderance of substitutions.

Nevertheless, the interpretation of these results should be done cautiously. The authors of the 2019 WMT report mention that *“the high fluency of neural translations induced the trained models to perform few reordering operations, leaving lexical choice as a main direction of improvement, as suggested by the larger amount of substitutions”* (Chatterjee et al. 2019 p. 23). An option to focus more on word substitution may not help improve the systems’ precision, if, for example, all insertions a system performed were wrong. Accordingly, maintaining a typical distribution of edit operations is a good indicator of a well-balanced APE system, but this is still a very coarse-grained evaluation, not allowing us to interpret the results in terms of language features.

The fact that all these automated forms of evaluation are insufficiently informative about what they actually mean in terms of the quality improvements produced by the APE systems called for human evaluation, which is the theme of the next section.

### Human evaluation

In 2016, it was decided to include a human assessment of the output of the APE systems, using DA, an evaluation process that is considered to provide reliable crowdsourced quality annotation. DA features a blind quality checking mechanism that controls reliability and consistency of annotations by crowdsourced resources. The annotators are asked to classify the adequacy (proximity of meaning to the source sentence) of different outputs, which are presented in a random order, combining APE outputs, MT outputs and human post-edits. The 2018 report describes the extent of this evaluation stage, as the two subtasks were evaluated by human professionals. This evaluation involved a question on the degree of adequacy (from 0 to 100) of a sequence of suggested translations for different source segments. A total of 64 hours were dedicated to this by 12 evaluators, and a total of 14,000 segment pairs of PBSMT content and 7161 pairs of NMT content were evaluated.

**Table 10:** Results of the human evaluation in all shared tasks

Year	2016	2017		2018		2019
Language pair	EN–DE	DE–EN	EN–DE	EN–DE	EN–DE	EN–DE
Best APE	AMU	FBK	FBK	MS/UE	FBK	UNBABEL
MT+APE	SMT	SMT	SMT	SMT	NMT	NMT
	+ NPE	+ NPE	+ NPE	+ NPE	+ NPE	+ NPE
Human PE	2.058	0.199	0.520	0.500	0.430	0.154
Best APE	0.867	0.040	0.261	0.410	0.240	0.056
Baseline MT	-0.499	− 0.008	− 0.083	− 0.220	**0.200**	− 0.054
**Upper distance**	**1.191**	**0.159**	**0.259**	**0.090**	**0.190**	**0.098**
**Lower distance**	**1.366**	**0.048**	**0.344**	**0.630**	**0.040**	**0.110**

Table 10 presents the average standardised scores achieved by the best APE systems in the human evaluation stage, together with those scores for the upper bound (the human post-edits) and the lower bound (the MT output). According to these scores, the only year in which the MT output obtained a positive result was in 2018, the first year NMT was present at this evaluation. The year in which the best APE system was closest to the human PE was 2018, when the MT output was SMT and the APE system was neural. 2016 was the year in which the best APE system was furthest from the MT output, and 2018, with NMT output and an NPE system, was the year with the shortest distance to the lower bound. In 2019, the APE system was almost equidistant to the lower and upper bounds.

These results show that APE is still somehow recognisable, a linguistic output that presents lower quality than human PE but better than raw MT. However, DA is also a limited form of evaluation, which focuses on one of the dimensions of translation quality—adequacy—which measures a subjective notion of how much of the meaning of the source is present in the candidate translation. The presence of previous examples is known to influence the results, and it has other limitations that the organisers of the shared tasks have tried to assess.

From the scientific point of view, this effort to improve the methods of evaluating APE should focus on the more informative forms of assessing its results. In shared tasks, it is important to use the best ranking methods, but that focus is not so useful outside of an environment with competing systems. Some of the evaluation methods used may work in a complementary way, describing with more detail what happens in improved and deteriorated sentences. It would also be beneficial if a more solid form of linguistic information was appended to the interpretation of results, which sometimes seems too speculative. Finally, the role of the human evaluation could also focus on answering some of the questions directly related to the behaviour of the systems. At this stage, we do not have, for example, knowledge on whether systems are better at inserting new words or deleting superfluous ones. Furthermore, although we may hypothesise that systems need to be better at dealing with lexical issues, do we have any evidence that the remaining problems are solved with word substitutions?

## Beyond APE

To improve the capability of APE systems, researchers used techniques that either extended the volumes of edited data available, or complemented the task with other approaches. In 2017, two such techniques were used and demonstrated their worth: the creation of synthetic data and resorting to features extracted by QE systems.

### Synthetic data

Post-edited data is scarce. To fulfil the data requirements of NPE, one solution is to resort to artificially-generated or synthetic corpora.

Sennrich et al. (2016b) showed that adding synthetic data to the training corpus of an NMT system improves the translation quality of the output. This first reference to the creation of synthetic examples of bilingual data started from a large volume of monolingual target data and created artificial source segments for this data by a “back-translation” MT process. This artificial bilingual data was then used as additional training data. The success of this strategy popularised it in MT training settings.

Inspired by their success, Junczys-Dowmunt and Grundkiewicz (2016) developed a training corpus for the APE task containing synthetic data. The data was generated via a 3-step process that included:

1. bootstrapping and filtering target-side (German) data to create language models for use in the next round-trip translation stage;
2. performing a round-trip translation process to create new artificial triplets;
3. another filtering pass, based on TER, to extract only high-quality translations that can be considered as PE data.

Next, a brief overview of the round-trip translation (step 2. above) is presented. As mentioned previously, APE requires triplets, so the simple creation of artificial segments to fill in the source side is not enough. For the experiment with an NPE system, Junczys-Dowmunt and Grundkiewicz required more EN-DE training pairs than what the organisers had made available. After having bootstrapped a fair volume of monolingual target data (DE) to extract language models for both languages, they used an MT system to translate part of this content, first to EN, and then back to DE. Their artificial triplets were then composed of:

- *SRC:* the EN MT outputs;
- *MT:* the DE strings from the back-translation;
- *PE *: the DE data that was originally in the monolingual reliable reference data (and which was the original source for the whole process).

This process proved to be very efficient (10 million new triplets were created in 24 h) and to have a positive impact as additional training data for NPE systems. This synthetic data is supposed to contain translations which are more literal than any reference translations that could be used, but it may enhance similarities with corrected PE data. The effect of this process may be a reinforcement of the editing patterns in the data, which might help the learning task, but in turn skew the data towards an overfitting effect.

The eSCAPE synthetic corpus, which is used as extra training data since the WMT18 shared task (see Sect. 2.4), was created by a different process. While the SRC and MT are artificial (created from machine-translating sentences in a real monolingual reference corpus) in the approach devised by Junczys-Dowmunt & Grundkiewicz, of the three components of the triplets in eSCAPE, the SRC and the PE come from real data and only the MT is machine-translated. However, this real data comes from available parallel corpora, meaning that it describes a translation process from source to target language, not a PE process over the MT output. Therefore, as the authors say, only the SRC is not a simulation of the PE process. This corpus contains 7.2 million triplets for EN-DE and 3.3 million of EN-ITA, translated first by PBSMT and then by NMT.

Freitag et al. (2019) tested the processes of round-trip and back-translation to train NPE models with monolingual synthetic data alone. The purpose was to reduce what the authors call “translationese”, the skewing in the output caused by the MT systems, thereby creating more “natural” output. The most interesting conclusions of the paper are the insights to the effects of the round-trip process: the round-trip translation data achieves higher BLEU scores and higher n-gram precision than the MT output, when compared to the reference translations, but both the MT output and the round-trip data have poorer vocabulary than the reference translations, an effect also noted by Vanmassenhove et al. (2019). The authors describe the results of round-trip translations as *“a less clean (paraphrased) version of the references, having been forward-translated from an already noisy back-translated source”* (Freitag et al. 2019 p. 41). The result of this NPE process is an improvement of the MT output, but there are no evidences or measures of the improvements in terms of “naturalness” of this output.

These experiments with synthetic data show their usefulness not only for training in research contexts, but also in actual uses of APE, where it can reduce the need for retraining systems with new data. However, the use of back-translation is not without risks, as discussed in Poncelas et al. (2018). Besides, the artificial nature of this data means that it cannot be used for evaluation. Since researchers need PE references to compare against the outputs of the APE systems, shared tasks and other evaluation initiatives still have to resort to real but scarce PE data to assess the achievements of their systems.

### Combining APE and QE

In 2017 and 2018, several papers and events explored the combined use of APE and QE. The first two papers presented here achieved the second- and third-highest ranks in the QE 2017 shared task (word-level) and the fourth- and fifth-highest ranks in the APE 2017 shared task.

Martins et al. (2017) describes Unbabel’s system that leverages QE features and APE outputs and applies them to a neural QE system. They use unigram, bigram and syntactic features that are composed of elements such as context, PoS tags, and syntactic relations, obtained using a specific tagger and parser tool. In their APE-based QE system, the APE task serves as a quality labelling component: after training the APE system, a TER alignment tool is used to classify the words in the PE sentences according to quality labels. They stack a linear and a neural model, and they use jack-knifing as a strategy to tackle overfitting. Their system seems to be able to achieve better QE predictions in longer sentences.

The system presented by Hokamp (2017) uses the same principles in a unified model for APE QE. QE is seen as a word-labelling task, based on language features that are useful for representations used by APE systems. So, QE features extracted during the first stages of the process are added as input factors to the APE system. The system is very effective in both tasks.

Chatterjee et al. (2018b) discuss different ways to combine APE with QE and highlight three strategies to apply QE scores in APE tasks:

- *QE as the activator of APE*: use sentence-level QE predictions on the raw output of an MT system to trigger its automatic correction when the estimated TER scores are below a certain threshold;
- *QE as a guidance for APE*: word-level binary quality labels are used to identify problematic words that should be corrected;
- *QE as a selector of APE output*: QE features are used to select the sentences and/or words that are the most accurate, between the output of APE and MT systems.

The first and the third strategies call for a lighter integration of APE and QE, since the output of the QE system is only integrated with APE either before or after the MT processing. The second strategy, however, requires a tighter integration with the MT system. The authors mention the different challenge this poses to an NMT system: where an SMT system easily accepts the addition of fine-grained information to the decoder, in NMT, a guided decoder needs to predict not only each word based on the previously predicted word, its context and its hidden state, but it also needs: (i) a method to prioritise the suggested word in the beam search; (ii) a look-ahead mechanism to avoid duplicates and (iii) a strategy to generate continuous and discontinuous target phrases.

The conclusions of the paper point to the word-level quality labels being more effective than sentence-level ones. In the evaluation conducted by the researchers, the strategies to use QE as a guidance and as a selector are the ones that show the best results. Finally, the authors suggest the development of end-to-end models that simultaneously leverage both technologies (APE and QE), using for example, pointer networks (Vinyals et al. 2015) for NPE.

The AMTA2018 conference also featured a workshop on the combination of APE and QE, which included participation from industry, system developers, and researchers (Astudillo et al. 2018).

The industry was represented by eBay and Booking.com, which showed the contribution of both APE and QE to their technology, but no combined systems. For eBay (Ueffing 2018), QE is applied to a Named Entity Recognition (NER) task, for the purpose of removing bad examples, and APE is still a research project, intended to become a second-stage correction system. For Booking.com (Khalikov 2018), QE is seen as a scoring system that selects good enough examples for publication and sends examples below that classification for editing; the inclusion of an APE component is still a planned strategy, aiming at replacing human editing. Booking.com also sees NER as the task for which these approaches may bring more valuable input.

System developers were represented by Unbabel (Graça 2018) and ModernMT (Federico 2018). Unbabel’s system uses QE as an indicator to human post-editors of the quality level of a sentence and of words that may need editing. ModernMT added a QE feature as part of their data cleaning process. For this team of researchers, APE is an option to be explored as a second stage, implemented on top of an adaptive system, but it may show a limited gain on top of a fully-fledged adaptive NMT system.

At this workshop, Junczys-Dowmunt presented a state-of-the-art reflection on how to combine APE and QE (Junczys-Dowmunt 2018b). In this presentation, he explained that one of the aims of APE is to explore the synergies of two technologies, namely adding NMT to an SMT system. He also mentions that APE and QE are just bug fixes that explore very narrow error margins, and that this technology does not allow exploitation of full error margins. He also presented a few words of caution, including reflections on the fact that current observations are being done with favourably chosen test sets, domains and language pairs, and he mentioned effects that simply come from combining systems and from two-pass decoding.

In 2019, the interest on joining the two applications seems to have faded. At the MT Summit, there was a workshop which built on the AMTA2018 one, with the title “Human-Aided Translation”^4^. Most of the presentations were on separate uses of the technologies, with only Turchi et al. (2019) showing the use of QE as an informer to the APE process. The suggestion is to use QE to produce an editing effort token. The conclusion of the paper, however, is that, in practice, this combined approach still needs to be improved, with more reliable QE predictions and more robust APE models.

## Neural post-editing tested in a commercial context

So far, this article has focused on academic experiments with APE, in shared tasks that have limited scope and controlled conditions. However, can APE be used in commercial contexts, and can it be useful in production workflows? In this section, we briefly comment on a project that tested and discussed the then state-of-the-art of APE in a commercial production setting.

### The 2018 ADAPT/Microsoft NPE project

Shterionov et al. (2020) present a joint project developed by the ADAPT Centre and Microsoft’s GSX Language Technology team in 2018. In this project, all content was made up of UI strings, containing data ranging from very short segments to segments with several sentences. These strings are drawn from different Microsoft software products, produced over several years with different SMT systems. The PE versions were always carried out on that MT output by human translators, using translation memories and other support resources. In the experiments, there was no use of synthetic data, since the available data was considered to be adequate: it included 200,000 segments for each of the language pairs (EN–DE and EN–ES). This enabled testing for real commercial settings.

There are several items which make this an innovative project. To the best of our knowledge, this was the first time that an APE strategy was applied to such a volume of real data, produced in a commercial environment. This was also the first time that the same source data was translated and tested for two different target languages, and the first time that training and test data had no length restrictions.^5^ Finally, as described below (see the description of “augmented” systems), this was also the first time that tokens identifying specific partitions in the data were used in APE experiments. All these factors placed specific challenges on the project, namely because of the need to demonstrate the reasonableness of investing on the implementation of an APE system in a production chain.

Shterionov et al. (2020) include a roadmap of the decisions to implement an APE system, from definition of the architecture to determining how the data is used in training. In the experiments, several neural systems and setup variables were tested. The ‘vanilla’ systems had different types of dictionaries (character, word, or BPE), while others differed in the input representation: single source (in which SRC and MT were concatenated), or multi-source, with SRC and MT fed separately. In addition, augmented systems were tested, which explored a novel modification of the APE systems: inspired by transfer learning for MT (Sennrich et al. 2016a; Johnson et al. 2017; Mattoni et al. 2017; Vanmassenhove et al. 2018), the developers inserted a token at the beginning of each segment which identified a specific partition of the dataset. This token either identified one of four length partitions (segments with less than 5 words, segments from 5 to 9 words, segments with 10 to 30 words, and segments with more than 30 words), the tenant (a group of projects, according to Microsoft), or the “TenantPartition” (a method which gathered less representative tenants in one single group). The addition of these tokens yielded the best results, although not the same for the two language pairs.

### Project results

The best-scoring systems were different for the two language pairs. While for EN–DE, the best system was the one which was augmented with the token that identified the “tenant partition”, in EN–ES, it was either the system augmented with the length (ranking by TER) or the system augmented with the tenant (ranking by BLEU). In any case, the improvements of applying an NPE system to SMT output outperformed any of the best systems presented in shared tasks. The scores and improvements are presented in Table  11.

**Table 11:** Evaluation scores in ADAPT/Microsoft project

Lanuage pair	System	BLEU (Multeval)	TER (Multeval)
EN–DE	Baseline: SMT	40.80	39.10
	NPE Augmented w/ TenantPartition	64.60	26.20
	**Improvement**	**23.80**	**12.90**
EN–ES	Baseline: SMT	60.10	25.60
	NPE Augmented w/tenant	66.50	22.90
	**Improvement**	**6.40**	**− 2.70**

These never-seen-before results in terms of TER and BLEU improvements were not repeated in terms of precision, since the best EN-DE system reached a precision of 62%, and the best EN–ES reached only 51%. We recall that the best APE system in WMT shared tasks, the system presented by Microsoft/University of Edinburgh in 2018 (Junczys-Dowmunt and Grundkiewicz 2018) achieved a precision of 68%, after having modified a similar percentage of sentences (75%). However, in the ADAPT/Microsoft project, the volumes of data were higher, with 10,000 segments in the test set. The percentage of over-correction in all these systems is still high, around 25% for EN-DE and 35% for EN-ES.

### Analysis of results

A detailed analysis of the results of the project showed that the best NPE models still struggle with very short or very long segments, segments with very low or very high initial TER(smt,pe), and segments with no errors, only one error, or higher numbers of errors. These different types of segments present different challenges, and the article describes some of these, like the effect created by BPE in one-word segments, or the presence of many untranslatable elements in long segments. Nevertheless, the competence of the NPE system in the majority of the segments clearly compensates for these difficulties, achieving very good global scores.

The distribution of edit operations also shows that the NPE systems developed in this project are well-balanced, showing a fair number of each edit. Still, we can see in Shterionov et al. (2020) that the number of substitutions is quite high (47%), when compared to the 40% in Junczys-Dowmunt and Grundkiewicz (2018). The latter project had a higher proportion of insertions (22%, against 15% in the ADAPT/Microsoft project). It is not clear yet whether these differences in the distribution of the edits reveal any effects of the performance of the systems.

The project included a test on the application of NPE to NMT output. The best systems for both language pairs achieved fair improvements in TER and BLEU scores, but it was considered that the NMT system was under-performing and further investigation was required.

This project represented an application of the state-of-the-art of APE in 2018, when neural systems were starting to become the norm in MT, but when no reasonable volume of post-edited NMT output existed. Still, the project gave an important contribution to the use of APE systems in commercial contexts.

## Open challenges for NPE

### The challenge of high-quality NMT output

The adoption of NMT systems, with the expected increase in quality of the output, created extra challenges for APE systems. Firstly, although it requires more time, NMT reduces the number of edits done by translators. (Sánchez-Gijón et al. 2019) This results in a narrower margin for edit learning. Secondly, NMT edited output may also show different types of errors that APE systems are not used to dealing with. This challenge began to surface in 2018, but researchers have not yet been able to present robust solutions to respond to it.

### Over-correction

A common difficulty in APE systems is application of the right number of corrections. APE systems tend to apply the editing patterns that were learned in new and unrelated contexts. Even the most recent systems show evidence of this ‘over-correction’: the best-scoring systems still deteriorate the TER scores in at least 20% of the segments that they modify (see Table 9).

### Generalisation

Another challenge to consider is the capacity of the systems to generalise and apply the results to new sets of data, be it in a different domain, or in content with different features, which may or may not have been represented during training. Most of the shared tasks have dealt with only one language pair. When a second pair is used, the amount of data, the domain and the quality were very different, which makes it hard to identify which of these factors is most influential. The ADAPT/Microsoft project confirms this, as the results were very different for the two language pairs.

The difficulty in generalising may have to do with overfitting: the dependence on the exact post-edits that were created because of the specificities of the MT output in the training data. If the dataset is too irregular, the APE system may not learn many edits; in contrast, if it is too regular, it may overfit. The best results in APE were obtained by the ADAPT/Microsoft project, when the dataset was composed of the same type of content, produced over several years and across different MT systems, but edited by professional translators following rigorous procedures. In ideal commercial settings, human translators have access to terminology and referential resources that allow them to maintain a strong consistency in their edits. Still, it is not clear whether these same results would be obtained by systems trained on this data, but translating different types of content.

### Data issues

NPE approaches are usually end-to-end systems, with no specific tools to handle specificities in the data. The papers reviewed in this article debate the effects of increased volumes of training data, but in this section we would like to focus on the importance of looking deeply into the features of edited data.

We may go back to the pilot APE shared task in 2015 to see a detailed analysis of data issues beyond the repetitiveness of the training data. Wisniewski et al. (2015) presented a system that tried to learn edits by using edit distances. Besides the over-correction effect mentioned earlier, the authors mention “uniqueness of edits” as a major issue: even the most frequent edits (e.g. insertion of Spanish punctuation) only describe a small percentage of the errors. Moreover, issues that translators need to correct frequently (like punctuation or case) are often neglected by APE. The authors trained a second system focused on specific errors, but the results were not satisfactory either, because of inconsistencies in the corrections. The ADAPT/Microsoft project (see Sect.  5) also included a brief analysis of most frequent edited tokens, but more details would be required to assess how to apply the findings to improve APE systems.

Other data features are still to be explored, such as the effect of segment length, the data domain (for example, UI strings are known to have specific features, like a high frequency of non-translatable elements, which may affect the results of APE systems), and there are other details related to the patterns of the edit operations themselves that deserve more analysis.

### Distribution of weight between inputs

Aside from improving MT outputs, researchers have invested in a better understanding of how APE systems work and in the interpretation of their results, namely in determining the added value of the obtained improvements. Jauregi Unanue et al. (2018) used a shared attention mechanism to identify the different contributions of the source side and the target side inputs of a contextual APE system, to the correction of the MT output. By using two separate encoders, one for the SRC input and another for the MT input, feeding a shared ‘flat attention’ mechanism, it was possible to analyse the separate distribution of weights for each input. A comparison of plots of attention matrices shows that the NPE system shifts the attention weights between the SRC and MT tokens at each decoding step. This also shows that, whenever there is a mistake, the model learns the correction by focusing on the SRC side, thus revealing the importance of contextual APE models, as opposed to monolingual ones.

### Combining APE and QE

Although there was a strong interest in the combination of APE and QE in 2018, there have not been many attempts at testing the use of these more complex settings. Since some of the most used NMT toolkits do not support features, the alternative has been to add tokens that contain information collected by QE modules to the APE training data. QE has also evolved into neural methods, which adds levels of difficulty in selecting and manipulating the effects of each feature. Because of this, it is still not clear yet whether the combination with QE is a promising avenue of research for APE.

### Evaluation metrics

One of the main purposes of the APE shared tasks has been to reflect on the best methods to evaluate the performance of APE systems. The evaluation reports from the shared tasks are well balanced, in terms of in-depth analyses of the information these methods provide and their limitations. However, for APE to be used in real scenarios, new metrics may need to be put in place.

There are two main purposes for the implementation of APE for commercial use: to increase the volume of MT content that may be published without any human post-editing, and to reduce the effort that is required to edit content that cannot be published ‘as is’.

A simple metric that accounts for the volume of content that passes above a specific quality threshold after APE is easily implementable. To measure the editing effort required for the rest of the content is not so easy. Academic metrics and methods have yet to prove their worth in commercial settings.

Beyond these, there is the need to evaluate the degree to which APE systems may help in production contexts. One way to measure this is in terms of productivity gains. Pal et al. (2016a) measured productivity by comparing the number of words produced per minute/hour by four translators, while post-editing raw MT output and post-editing the output of a multi-engine APE system. The average productivity gain is established at 12.96%, but the results, even with such a small sample, show a high variability, from a loss of 40% productivity (from a user that perhaps should have been excluded from the study, due to having interrupted the task for a long time during the evaluation) to a gain of 46.6%, with the other values being 5.0% and 33.3%. Other publications, like Ortega et al. (2019), refer to productivity gains, but do not include them in the evaluation. So, claims of increased productivity thanks to APE are yet to be tested. Furthermore, the evaluation of usefulness of APE systems in production may require a more fine-grained evaluation. For example, if an APE performs mostly substitutions, are these consistent, as required in production? Or will APE systems insert random words, and force post-editors to perform extra work, checking that the same terms are used consistently in different projects?

### The use of APE in production workflows

APE research has not paid much attention to its relation with human PE, although reducing the PE effort is one of the intended uses of this task (see Sect. 1). In this section, we analyse a few papers that cover this theme.

The main tools used by professional translators are translation memories (TM), databases of previously translated pairs of segments which are later retrieved according to the degree of similarity to new translatable segments. ‘Fuzzy matches' represent different levels of similarity between a segment in the TM and the new segment, and which may require varying degrees of editing. There have been suggestions to develop automatic assisted systems to edit these segments by implementing methods of example-based MT (Kranias and Samiotou 2004). More recent explorations of this application of APE involved the combination of methods to repair fuzzy matches with an APE component, in papers like Knowles et al. (2018) or Ortega et al. (2019). The quality gains claimed by these papers seem to reveal the validity of the combination of MT and TM, but only Ortega et al. (2019) included human evaluation, performed by one single person, who was not a professional translator. Both papers suggest that further research in this area is necessary, also exploring the eventual contribution of QE methods.

Another concept in close proximity to the combination of MT and TM is the development of interactive MT systems. The first systems to experiment with interactive APE were related to SMT technologies (Simard and Foster 2013), some of these integrating online learning (OL) to feed adaptive suggestions to the PE process (Ortiz-Martínez and Casacuberta 2014; Lagarda et al. 2015). Chatterjee et al. (2016b) and Chatterjee et al. (2017b) describe two moments in the evolution of an OL APE system that is capable of choosing the best corrections from data from different domains. Furthermore, it aims at fulfilling the other role of an OL system: to collect editing data from the human post-editor, incorporating this in real time to its training data. However, the processing and evaluation of these systems do not involve human post-editors. The PE process is simulated, step by step, in a sequence which learns incrementally from the corrections made to the test data. A fundamental element in this online process is the instance selection mechanism, which decides on-the-fly if it has enough information to select the best corrections, from a stream of data points that come from different domains, or of it should simply not edit a segment. The results show that this mechanism improves the precision and reduces the typical over-correction of APE systems. In the second paper, the authors add a negative feedback mechanism, which allows for a broader record of the word and phrase-level corrections and the contexts in which they occur, than simply saving the corrected segments. This seems to allow the system to learn how to correct its own mistakes. These papers contain valuable insights on the use of APE in production scenarios in which generic multi-domain data is used to translate domain-specific texts.

Negri et al. (2018a) apply OL APE systems to NMT output, and highlight the usefulness of such systems to eliminate the need for fine-tuning or retraining MT systems from scratch, while seamlessly integrating this process within production systems. The use of post-editor’s knowledge should not only improve the quality of the outputs but also the quality and productivity of the human post-editors. The training of the APE models includes synthetic data, and the simulated experiments aim at representing different degrees of complexity, covering a gamut of real case scenarios. The results of the experiment point to real advantages of the incremental learning of OL APE systems, namely in the demanding task of improving already high quality NMT output. However, there is a limit to the capacity of this learning, and the authors question the balance between the costs of investing in MT development and integrating this form of continuous adaptation into production environments. A realistic analysis of this conflict seems to point to APE being a reasonable strategy for budget-constrained translation production outlets, but the real proof of applicability of APE with interactive learning features to human production is an area that requires more research, with human input and evaluation, ideally in real production scenarios.

## Conclusions: the role of APE

This review of the state-of-the-art of APE covers a period until the end of 2019, describing 5 years of rapid evolution of a dynamic research area. The results achieved by APE systems in the most important experiments in this period produced extreme reactions: some pessimistic about the future of this technological approach and others perhaps too optimistic.

Although short, the history of APE is a rich one. In the evolution of APE models from SMT to NMT, some of the approaches that had been explored were left behind: with end-to-end solutions, systems stopped focusing on language details; the incorporation of QE features, although promising in 2017, was not explored in 2018 or 2019; monolingual models, although at the root of the best systems in 2016 and 2017, were replaced by context-aware features provided by the combination of multi-encoders and multi-attention systems. The use of BERT in 2019 to develop sentence representations and enable efficient concatenation of multiple sources may represent an innovative application of technologies in the context of NPE, and appending informative tokens to multi-source inputs also seems to be a promising strategy in this context. Nevertheless, despite indications to the contrary, APE still seems to be alive and kicking.

When the death of a technology, or of a technological approach to a problem, is announced, these statements are usually founded on a linear view of the evolution of technology. However, as we have seen so often, technology and knowledge evolve in a cyclical way, with previous approaches coming back when recent methods reach a plateau, and researchers need to revisit solid and tested ideas. The short history of APE is one of rapid evolution, especially in periods of transition between technologies: first, from RBMT to SMT, and then from SMT to NMT. This second cycle began with pilot experiments, in which SMT techniques were applied in a second step to SMT decoders. The challenge which APE faces now is similar to that initial one: researchers are trying to improve a system by digging into the details of the same technology that was used in the first decoding stage. Although the error margins are narrow, and improvements are not comparable to those identified in technology-transition periods, the value of the findings in that research effort should not be dismissed. It is also important to stress that NMT is not a solved problem either: when researchers work within specific, real-life scenarios, it becomes clear that there is no ‘one-solution-fits-all’ and often the gains are not impressive. For that reason, research into the details of NMT is necessary. APE could, after all, be an excellent way to bring some of the most important dimensions in NMT to the surface, when this technology is applied to demanding use-cases in which only small improvements are possible.

We described in the introduction (Sect. 1) some of the possible uses set out for APE. With NPE end-to-end approaches, some of those purposes cannot be fulfilled. For example, in NPE systems we do not yet have access to deeper text analyses, or systems are not focused on systematic types of errors. Maybe revisiting those specific analyses would bring back the type of discovery that makes APE a challenging but worthwhile research effort. Notwithstanding these issues, NPE can still fulfil the third purpose presented in that section of the introduction: to improve the output for human PE.

In that same section, we introduced three questions that APE projects should try to respond to. While we can say that APE projects have looked carefully into the relation between the original MT output and the APE results, and that the evaluations and descriptions of competing systems have shed light on systems that, without scoring very high in the rankings, included promising technological takes on the APE problem, the capacity of APE systems to deliver consistent results across different language pairs has not yet been achieved. This shortcoming has been associated with the quality of the MT output (high TER scores in the original MT output), but it is not clear why, for example, different languages have such different scores.

APE is an environment for testing techniques that come from other areas. Some of the techniques that may be further explored to improve APE are related to methods on how to: add annotated data (e.g. using PoS tags); include different types of features (e.g. from QE); adjust APE systems to different language pairs, and different edit distances, based on the training/development data; balance repetitiveness and representativity in the training data; handle specific editing patterns; analyse the impact of partitioning the data according to its relevant characteristics or specific use-cases; deal with long segments, which are common in industrial contexts, and develop effective methods to process them into smaller units; or to analyse the impact of synthetic data in exacerbating learning patterns in the training data. Any of these approaches may bring relevant findings, with impact on other areas of research. One of the areas in which APE has proved its worth is in focusing on the data features that can make a difference in the demanding narrow-margin improvements that NMT cannot solve. The use of synthetic data, the analysis of repetitiveness, and the focus on process data, like edit operations, are some of the tools that APE provides to analysing such challenges. In addition, further study is required on the variable options in constructing an APE system, e.g. encoder/decoder architectures, word-segmentation for input/output vocabularies, etc., which may have a high impact on the quality of the APE system.

The state-of-the-art of APE in 2019 is composed of systems that thoroughly exploit the capacities of NMT technologies, through system combination and ensembling, multi-source training, Transformer models, synthetic data training, fine-tuning with BERT, token appending, and different strategies to mitigate over-correction. In WMT shared tasks, it was possible to see how these approaches have handled increasingly difficult challenges. Outside of these events, there has not been enough research on applications of APE to guarantee that commercial projects and investments will always achieve a high return, or that they will always break new records of performance.

The ADAPT/Microsoft project has shown that APE can be used to good effect in a context in which companies deal with a transition between technologies. In such contexts, companies have to consider whether to discard legacy data or to try and learn from it, while they still do not have sufficient and consistent enough data built from the new technologies. This is an excellent example of a successful application of APE: by learning editing patterns from legacy data, APE can be integrated into an existing workflow, adding value to existing data, and bringing it forward, without needing to replace the whole translation technology which is in place.

However, there are other useful applications of APE, even when a powerful technology has replaced the previous one. In corporate environments, APE applied within the same technological paradigm (like NPE on NMT output) can be used to:

- *reduce the amount of (re)training*: implementing NPE as a secondary system would reduce the necessity of retraining MT systems, when new data is made available and researchers want to keep control over different stages of the process;
- *multiple APE for specific issues*: different APE systems can target specific issues and be trained on small amounts of data that cover these issues, to complement stable corporate-wide MT systems;
- *domain adaptation*: APE can act as an alternative method to domain adaptation, or a good application of transfer learning, when a model trained on a specific set of data tries to improve the output of another system (this can be tested, for example, within the IT domain, between such different text types as UI—highly fragmented and uncontextualised—and documentation, manuals, and other types of more structured text);
- *explore monolingual data*: post-edits that dynamically come into a production workflow can be used to gradually and constantly improve MT outputs;
- *a complement to QE*: APE can be used on top of QE systems, to reduce the risk of serious errors being present in the MT content deemed as publishable, working exactly within the small margin of improvement that is required in such circumstances;
- *develop online learning interactive systems*: APE’s use as an interactive personalisation tool, which learns specific edits and reapplies them in the course of a work sequence, is an application with potential interest;
- *aiding new metric development*: APE can help develop new metrics for the use and reuse of MT output in dynamic commercial workflows, by measuring specific properties of the post-edited sentences, or contributing to the evaluation of the usefulness of APE at a document level.

NMT methods may have had a strong impact on our perception of APE’s usefulness. Indeed, NMT helped redefine APE, or guided it into new directions, but this did not necessarily imply its demise. As a special-purpose learning phase, or a second-pass decoding stage, APE cannot simply be a redundancy process, but it may be a useful complementary step in demanding translation production environments. It continues to serve the purpose of bringing new light to legacy and recent post-edited data, and also to help us understand the components of translation technologies that make the big difference of the small details.

## Footnotes

1 Zero-shot MT and unsupervised MT are two MT approaches that do not explicitly require parallel data between source and target languages. However, they too exploit some parallel data, e.g. backtranslated data for unsupervised MT.

2 https://kantanmt.com/documents/GENTRY_File_Parsing.pdf

3 https://cordis.europa.eu/project/rcn/194324/en

4 https://sites.google.com/unbabel.com/hat19/home

5 It is important to note that, although the datasets contained segments with unrestricted lengths, to avoid memory issues at training time, these were trimmed to a maximum length of 300 tokens, a lot more than the usual 30 tokens. However, this limit was not imposed during translation, and there are examples of translated sentences with more than 300 tokens.
